# Lever Insertion as a Salient Stimulus Promoting Insensitivity to Outcome Devaluation

**DOI:** 10.3389/fnint.2017.00023

**Published:** 2017-09-27

**Authors:** Youna Vandaele, Heather J. Pribut, Patricia H. Janak

**Affiliations:** ^1^Department of Psychological and Brain Sciences, Krieger School of Arts and Sciences, Johns Hopkins University, Baltimore, MD, United States; ^2^The Solomon H. Snyder Department of Neuroscience, Johns Hopkins School of Medicine, Johns Hopkins University, Baltimore, MD, United States

**Keywords:** habit, goal-directed, stimulus-response association, satiety, devaluation, conditioned taste aversion

## Abstract

Flexible and efficient decision-making in complex environments can be achieved through constant interactions between the goal-directed and habitual systems. While goal-directed behavior is considered dependent upon Response-Outcome (R-O) associations, habits instead rely on Stimulus-Response (S-R) associations. However, the stimuli that support the S-R association underlying habitual responding in typical instrumental procedures are poorly defined. To resolve this issue, we designed a discrete-trials procedure, in which rats must wait for lever insertion and complete a sequence of five lever presses to obtain a reward (20% sucrose or grain-based pellets). Lever insertion thus constituted an audio-visual stimulus signaling the opportunity for reward. Using sensory-specific satiety-induced devaluation, we found that rats trained with grain-based pellets remained sensitive to outcome devaluation over the course of training with this procedure whereas rats trained with a solution of 20% sucrose rapidly developed habit, and that insensitivity to outcome devaluation in rats trained with sucrose did not result from a bias in general satiety. Importantly, although rats trained with pellets were sensitive to satiety-induced devaluation, their performance was not affected by degradation of instrumental contingency and devaluation by conditioned taste aversion (CTA), suggesting that these rats may also have developed habitual responding. To test whether the discrete-trials procedure biases subjects towards habitual responding, we compared discrete-trials to free-running instrumental responding, and found that rats trained with sucrose in a fixed-ratio 5 (FR5) procedure with continuous presentation of the lever were goal-directed. Together, these results demonstrate that discrete presentations of a stimulus predictive of reward availability promoted the formation of S-R habit in rats trained with liquid sucrose. Further research is necessary to explain inconsistencies in sensitivity to outcome devaluation when rats are trained with grain-based pellets.

## Introduction

Flexible and efficient decision-making in complex environments can be achieved through constant interactions between the goal-directed and habitual systems. While a goal-directed system allows for the consideration of actions’ consequences, and flexible adaptation when those consequences change, habits are automatically triggered by antecedent predictive stimuli and do not require the anticipation of the outcome of each available action. In associative learning theories, goal-directed behavior has been defined as dependent upon Response-Outcome (R-O) associations, the response being mediated by the expectation of the outcome (the desired goal). Since goal-directed responding relies on the causal relationship between the response and the outcome, and the current motivational value of that outcome, the performance decreases when the instrumental contingency is degraded or when the value of the outcome is reduced (Dickinson, 1994; Balleine and Dickinson, 1998). Habits, on the other hand, are defined by Stimulus-Response (S-R) associations and are not affected by outcome devaluation or degradation of the R-O contingency (Dickinson, 1994; Balleine and Dickinson, 1998).

It is well known that schedules of reinforcement can bias response strategy. Random-ratio (RR) schedules have been shown to promote goal-directed behavior whereas overtraining or random-interval (RI) schedules promote the formation of habits (Adams, 1982; Dickinson et al., 1983). Dickinson et al. (1983) suggested that after overtraining or under RI schedules, uncoupling of response rate and reinforcement rate would facilitate habitual learning, while the strong contingency between response rate and reinforcement rate in RR would result in goal-directed behavior. More recently, it was suggested that habitual performance under RI schedules was instead related to uncertainty and lower temporal contiguity between responses and reward delivery (Derusso et al., 2010). The dissociation between RR and RI reinforcement schedules illustrates one important aspect of rodent models of habit. Although habitual responding is defined as a S-R association, it is typically operationalized as an absence of outcome representation (Vandaele and Janak, 2017). Thus, training procedures that weaken the R-O association by reducing the contingency or the contiguity between the response and the outcome (e.g., RI schedules), are expected to produce a behavior less sensitive to the representation of the outcome. However, habits formed through these schedules may not appropriately model the transition from R-O to S-R control of performance that accompany long-term S-R-O experiences with extended training. In fact, it is questionable whether there have been direct demonstrations of habitual responding as an S-R association in rodents.

The demonstration of habit as a positive result, i.e., the reliance on an S-R association, is challenging because the stimuli supporting the S-R association, and thus habitual performance, are poorly defined in instrumental procedures. These stimuli are situational cues being associated with the instrumental response through repeated reinforcement. As previously highlighted, the incidental nature of this association make the stimuli difficult to identify and manipulate (Corbit and Janak, 2016b). Yet, these stimuli can considerably influence instrumental responding (Rescorla and Soloman, 1967; Holland, 2004; Thrailkill and Bouton, 2015). For example, Pavlovian stimuli predictive of an outcome can increase responding for this same outcome, an effect known as Pavlovian to instrumental transfer (PIT; Estes, 1948; Rescorla and Soloman, 1967; Lovibond, 1983), and the magnitude of this PIT effect grows with extended training and is not affected by outcome devaluation (Rescorla, 1994; Holland, 2004; Corbit and Janak, 2016a). Recent studies in humans have shown that presentation of a Pavlovian cue previously associated with an outcome is sufficient to trigger responding for that outcome, despite its devaluation by sensory-specific satiety (Watson et al., 2014; van Steenbergen et al., 2017). These results suggest that the PIT effect can promote cue-driven habit-like responding and counteract the goal-directed behavior otherwise observed in absence of the cue (Watson et al., 2014). However, the PIT procedure does not allow study of the formation of an S-R habit, since the stimulus and the response are associated with the outcome in separate phases of training.

In previous studies using a discrimination task in which stimuli predicted reward delivery upon execution of the appropriate response, rats over-trained in the task developed habitual responding (Faure et al., 2005, 2010) despite the use of double R-O associations, known to promote goal-directed behavior (Colwill and Rescorla, 1985; Colwill and Triola, 2002; Holland, 2004). These studies suggest that providing discriminative stimuli facilitates the expression of habit. Yet, this effect was not directly demonstrated. The objective of this study was to determine whether providing a salient discrete stimulus, predictive of reward availability, during instrumental training would promote the formation of S-R habits. To this end, we designed a discrete-trials procedure, in which rats must wait for lever insertion and complete a sequence of five lever presses to obtain a reward (20% sucrose or grain-based pellets). Lever insertion thus constituted an audio-visual stimulus signaling the availability of the reward. In addition, lever retraction at the completion of the fixed-ratio 5 (FR5) directly signaled reward delivery. Rats were trained under FR5 in this discrete-trials procedure to study sequence learning and investigate how parameters of automaticity (latency to first lever press, within-sequence response rate, within-sequence port entries) may correlate with the formation of habits. Sensitivity to outcome devaluation was assessed after different lengths of training using sensory-specific satiety. We predicted that the discrete-trials FR5 procedure, by providing salient stimuli signaling reward availability and delivery (lever insertion and retraction), would promote habitual responding through the formation of S-R association.

## Materials and Methods

### Subjects

A total of 73 experimentally naive Long–Evans rats (Harlan, IN, USA; 69 males, 4 females) weighing on average 300–330 g (males) and 220–240 g (females) at the start of training were individually housed and maintained in a light- (12-h light-dark cycle, lights ON at 7 am) and temperature-controlled vivarium (21°C) with partial enrichment. All experiments were performed during the light cycle. Rats were maintained at 90% of their free-feeding weight and food rations were given 1–2 h after daily behavioral sessions. Water was available *ad libitum*. This study was carried out in accordance with the recommendations of the Guide for the Care and Use of Laboratory Animals (Institute of Instrumental Training Laboratory Animal Resources, Commission of Life Sciences, National Research Council, 1996). The protocol was approved by the institutional animal care and use committee of Johns Hopkins University.

### Apparatus and Instrumental Training

Training and testing occurred in conditioning chambers housed within sound-attenuating boxes (Med Associates, St. Albans, VT, USA). Rats underwent a single 30-min magazine training session, in which a reward (0.1 mL aliquots of sucrose 20% or grain-based pellets) was delivered under a variable time-60 s schedule into a recessed magazine in the middle of the right wall of the chamber. Rats were next trained to press the left lever to earn a small aliquot of sucrose 20% (0.1 mL delivered over 3 s) or a single 45 mg grain-based pellet (Bioserv Biotechnologies), delivered in the adjacent magazine. Both rewards were delivered in an identical oval tray mounted in the magazine. Sessions ended after rats had earned a maximum of 30 rewards or 1 h had elapsed. The house-light, located on the ceiling of the operant chamber, remained illuminated during the full length of the session. After five sessions of this continuous reinforcement schedule (CRF), the discrete-trials procedure was introduced. Each session comprised 30 trials separated by 1-min inter-trial intervals. Every trial started with the insertion of the left lever. For the first two sessions, one lever press simultaneously resulted in the retraction of the lever, reward delivery, and the initiation of a new inter-trial interval (discrete-trial fixed ratio (FR) 1; DT1). The response ratio was then increased to five (discrete-trial FR5; DT5). Failure to complete the ratio within 1 min was considered as an omission and resulted in lever retraction and initiation of a new inter-trial interval. Sensitivity to outcome devaluation was tested using sensory-specific satiety after various lengths of training. Animals failing to learn instrumental responding after five sessions of CRF (*n* = 4) or whose performance fell below two completed ratios for three consecutive sessions before a test (*n* = 2), were excluded.

### Outcome Devaluation by Sensory-Specific Satiety

To avoid neophobia, rats were exposed to the control reward for 30 min in feeding cages 1 or 2 days before the 1st devaluation test. Each rat received 2 days of testing, separated by one reinforced training session. On the first test day, half of the rats were given free access to their training reward (either pellets or sucrose; devalued condition), while the other half received the control (alternative) reward (either pellets, sucrose, or maltodextrin, depending on the experiment; valued condition). Pre-feeding occurred for 1 h in feeding cages in the experimental room. Immediately after pre-feeding, rats were placed in the operant chambers for a 10-trial extinction session. The procedure was identical to that of training except that no reward was delivered. As in training, the lever extended at the start of each trial and retracted after 1 min or at the completion of the ratio. On the second test session, animals were pre-fed with the alternative reward prior to a second 10-trial extinction test.

### Degradation of Contingency

One day prior to exposure to the contingency degradation procedure, rats trained to respond for sucrose or pellets on the DT5 schedule underwent a 10-trial extinction test to assess baseline reward seeking (“pre-test”). The next 3 days, rats underwent contingency-degradation training, followed by a final 10-trial extinction test to assess post-degradation reward seeking. Contingency-degradation sessions consisted of 10 reinforced trials and a free reward delivery during each inter-trial interval (variable time-30 s). Thus, the probability of receiving a reward after the completion of a five lever presses sequence decreased to *p* = 0.5. To assess individual sensitivity to contingency degradation, we compared instrumental responding in the “pre-test” and “post-degradation” extinction sessions.

### Conditioned Taste Aversion

Rats were assigned to “paired” and “unpaired” groups for taste aversion training such that baseline response rate, and previous sensitivity to outcome devaluation, were balanced across the two groups, for each outcome type. Conditioned taste aversion (CTA) was induced in the animals’ home cages over two cycles of 2 days. On the first day of each cycle, rats in the paired group received 10 min access to their training reward whereas rats in the unpaired group received nothing. Immediately after this 10 min period, all rats (both paired and unpaired subjects) received an intraperitoneal injection of a 0.3 M lithium chloride (LiCl) solution (6 mL/kg). On the second day of each cycle, rats in the unpaired group received 10 min access to their training reward but none of the rats received an injection. Thus, exposure to the reward and LiCl injections was identical for the two groups but only rats from the paired group were subjected to LiCl-induced illness paired with reward consumption. To confirm the induction of CTA for paired rats and assess the level of generalization from the home cage to the experimental chamber, rats were exposed to their training reward in the magazine of the experimental chamber for 5 min. The magazine food cup was filled with 3 mL of sucrose or 25 grain-based pellets. On the test day, rats were then placed in their experimental chambers for a 10-trial extinction session identical to that described above.

### Data Analysis

Lever presses and port entries were collected throughout training and test. In the DT5 procedure, mean response rate was expressed in responses per second and was measured as the number of lever presses per trial divided by the time of lever availability, averaged across trials. Time of lever availability was set to 60 s in omission or incomplete trials. To compare change in sucrose and pellets consumption during taste aversion learning in Experiment 4, consumption in the home cage was expressed as the percentage of the amount eaten prior to treatment. In the operant box, consumption was expressed as the percentage of presented food that was consumed.

Four female rats were used in the last experiment. There were no main effects of sex or devaluation by sex interactions in this experiment. Analyses were thus conducted on pooled data. Notably, when data of males only were examined, the findings remained the same. Note that the small sample size of female rats precludes any strong conclusions regarding sex differences in habit formation. All data were subjected to repeated measures analyses of variance, followed by *post hoc* comparisons when indicated, using Tukey’s Honest Significant Difference (HSD) test. Significance was assessed against a type I error rate of 0.05.

## Results

### Rapid Development of Insensitivity to Outcome Devaluation by Specific Satiety in a Discrete Trials Procedure

**Figure 1:**
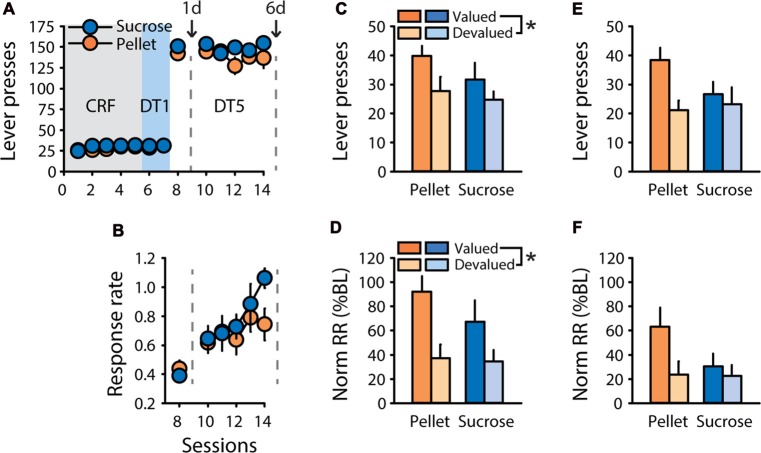
Rapid development of habit-like behavior in rats responding for sucrose or grain-based pellets in a discrete-trials procedure. Training and satiety-induced outcome devaluation tests in the discrete trial 5 (DT5) procedure in rats trained either with pellets (*n* = 7) or a 20% sucrose solution (*n* = 6). **(A)** Mean number of lever presses (±SEM) across training under continuous reinforcement (CRF, five sessions), discrete-trials fixed-ratio 1 (DT1, two sessions) and discrete-trials fixed-ratio 5 (DT5, six sessions) schedules in rats trained with grain-based pellets (orange circles) or sucrose (blue circles). **(B)** Mean response rate in responses per second (±SEM) across training under DT5 in rats trained with pellets (orange circles) or sucrose (blue circles). Gray dotted lines in **(A,B)** indicate the time of devaluation tests after 1 day (1d) and 6 days (6d) of DT5 training. **(C)** Mean number of lever presses (±SEM) in valued (dark color) and devalued (light color) conditions, at 1d test in rats trained with pellets (orange bars) or sucrose (blue bars). Main effect of devaluation: *p < 0.05 devalued compared to valued. **(D)** Mean normalized response rate (±SEM) in valued (dark color) and devalued (light color) conditions, at 1d test in rats trained with pellets (orange bars) or sucrose (blue bars). Main effect of devaluation: *p < 0.001 devalued compared to valued. **(E)** Mean number of lever presses (±SEM) in valued (dark color) and devalued (light color) conditions, at 6d test in rats trained with pellets (orange bars) or sucrose (blue bars). **(F)** Mean normalized response rate (±SEM) in valued (dark color) and devalued (light color) conditions, at 6d test in rats trained with pellets (orange bars) or sucrose (blue bars).

To assess the presence of sensitivity to outcome devaluation after training in a “discrete trials” (DT) procedure, designed such that each trial is initiated by lever insertion, rats trained to respond for a 20% solution of sucrose (*n* = 6) or grain-based pellets (*n* = 7) underwent satiety-based outcome devaluation after one or six sessions of DT5 training. After pre-training (see “Materials and Methods” Section), rats rapidly learned to press the lever for sucrose or pellets in the DT5 procedure, with the number of lever presses near the maximum possible from the first DT5 session (Figure 1A). Mean response rate increased during training as indicated by a main effect of session (*F*_(5,55)_ = 17.14, *p* < 0.0001), but did not differ based on the training reward (Figure 1B, *F*_(1,11)_ = 0.60, ns). Sensitivity to satiety-induced devaluation of the outcome was tested after one and six DT5 training sessions, using 20% maltodextrin and 20% sucrose as control rewards (pre-feeding in valued condition) for rats trained with 20% sucrose and grain-based pellets, respectively. After 1 training session, rats were sensitive to outcome devaluation, as indicated by a main effect of devaluation on lever pressing (Figure 1C, *F*_(1,11)_ = 8.59, *p* < 0.05) and on normalized response rate (Figure 1D, *F*_(1,11)_ = 22.94, *p* < 0.001). After five additional training sessions, responding was no longer significantly affected by outcome devaluation, as indicated by similar number of lever presses (Figure 1E, *F*_(1,11)_ = 4.13, ns) and similar normalized response rate (Figure 1F, *F*_(1,11)_ = 2.74, ns) in the valued and devalued conditions. Although there were no effects of reward nor reward by devaluation interactions, rats trained with pellets seemed more sensitive to satiety-induced devaluation than rats trained with sucrose, particularly after six training sessions.

### Insensitivity to Outcome Devaluation by Specific Satiety in the Discrete Trials Procedure Develops in Rats Responding for Sucrose Reward but Not Pellet Reward

**Figure 2:**
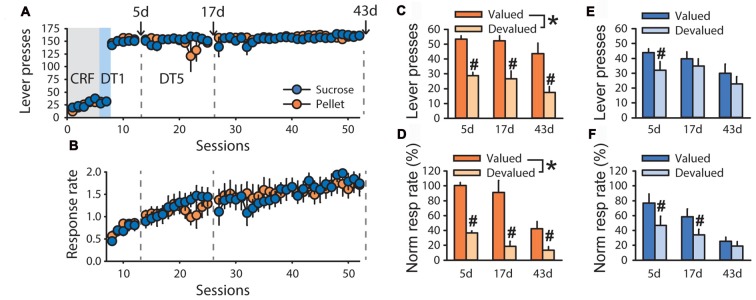
Time-course of habit-like behavior in rats responding for sucrose in a discrete-trials procedure. Training and satiety-induced outcome devaluation tests in the DT5 procedure in rats trained either with pellets (*n* = 5) or a 20% sucrose solution (*n* = 5). **(A)** Mean number of lever presses (±SEM) across training under (CRF, five sessions), discrete-trials fixed-ratio 1 (DT1, two sessions) and discrete-trials fixed-ratio 5 (DT5, 43 sessions) schedules in rats trained with grain-based pellets (orange circles) or sucrose (blue circles). **(B)** Mean response rate in responses per second (±SEM) across training under DT5 in rats trained with pellets (orange circles) or sucrose (blue circles). Gray dotted lines in **(A,B)** indicate the time of devaluation tests at 5d, 17d and 43d. **(C)** Mean number of lever presses (±SEM) in valued (dark color) and devalued (light color) conditions, across tests at 5d, 17d and 43d, in rats trained with pellets. *p < 0.01 devalued compared to valued across tests. ^#^*p* < 0.01 at 5d, *p* < 0.05 at 17d, *p* < 0.01 at 43d. **(D)** Mean normalized response rate (±SEM) in valued (dark color) and devalued (light color) conditions, across tests at 5d, 17d and 43d, in rats trained with pellets. *p < 0.001 devalued compared to valued across tests. ^#^*p* < 0.01 at 5d, 17d and 43d. **(E)** Mean number of lever presses (±SEM) in valued (dark color) and devalued (light color) conditions, across tests at 5d, 17d and 43d, in rats trained with sucrose. ^#^*p* < 0.01 at 5d. **(F)** Mean normalized response rate (±SEM) in valued (dark color) and devalued (light color) conditions, across tests at 5d, 17d and 43d, in rats trained with sucrose. ^#^*p* < 0.01 at 5d and 17d.

To explore possible differences in outcome sensitivity in sucrose- and pellet-trained rats, an additional experiment with a longer time course was conducted. In devaluation tests of the second experiment, grain-based pellets and sucrose 20% were used as the alternative control rewards for rats trained with sucrose 20% (*n* = 5) and grain-based pellets (*n* = 5), respectively. Despite similar performance during training, both in term of lever presses (Figure 2A) and response rate (Figure 2B, *F*_(1,70)_ = 0.0857, ns), sensitivity to satiety-induced devaluation differed as a function of the training reward (Figures 2C–F). Repeated measure analysis of variance (ANOVA) with tests and conditions as within-subject factors and training reward as a between-subject factor, revealed that lever pressing and normalized response rate were lower in the devalued condition compared to the valued condition at each of the three tests (main effect of devaluation on lever presses *F*_(1,8)_ = 33.59, *p* < 0.001, normalized response rate *F*_(1,8)_ = 49.27, *p* < 0.001). However, this devaluation effect interacted with the training reward (lever press *F*_(1,8)_ = 9.41, *p* < 0.05, normalized response rate *F*_(1,8)_ = 10.49, *p* < 0.05). *Post hoc* analyses of these interactions revealed that lever pressing and normalized response rate for pellets remained sensitive to devaluation across tests (Figures 2C,D, *p* < 0.01) whereas lever pressing and normalized response rate for sucrose were not affected by outcome devaluation (Figures 2E,F, ns). Accordingly, analysis of response rate across trials at each of the three tests revealed a main effect of devaluation for animals trained with pellets (Supplementary Figures S1A–C, test 1: *F*_(1,4)_ = 62.39, *p* < 0.01, test 2: *F*_(1,4)_ = 22.94, *p* < 0.01, test 3: *F*_(1,4)_ = 11.79, *p* < 0.05) but not for animals trained with sucrose (Supplementary Figures S1D–F). There was a significant effect of test (Figures 1C–F, lever press, *F*_(2,16)_ = 8.18, *p* < 0.01, normalized response rate, *F*_(2,16)_ = 22.02, *p* < 0.0001) but no devaluation by test (lever press, *p* = 0.97, normalized response rate, *p* = 0.07), reward by test (lever press, *p* = 0.79, normalized response rate, *p* = 0.97) or devaluation by reward by test (lever press, *p* = 0.40, normalized response rate, *p* = 0.65) interactions.

To assess the time course of the development of insensitivity to satiety-induced devaluation in rats trained with sucrose, a repeated measure ANOVA was conducted on each test separately. This analysis revealed a main effect of devaluation on tests 5d, 17d and 43d for lever pressing (Figures 2C,E, 5d: *F*_(1,8)_ = 22.08, *p* < 0.01, 17d: *F*_(1,8)_ = 12.82, *p* < 0.01, 43d: *F*_(1,8)_ = 24.73, *p* < 0.01) and normalized response rate (Figures 2D,F, 5d: *F*_(1,8)_ = 16.52, *p* < 0.01, 17d: *F*_(1,8)_ = 18.59, *p* < 0.01, 43d: *F*_(1,8)_ = 16.86, *p* < 0.01). This devaluation effect interacted with the training reward on lever pressing at 17d and 43d (Figures 2C,E, 17d: *F*_(1,8)_ = 6.42, *p* < 0.05, 43d: *F*_(1,8)_ = 7.78, *p* < 0.05) but not at 5d (5d: *F*_(1,8)_ = 2.34, ns). The devaluation by training reward interaction was only significant at 43d considering normalized response rate (Figures 2D,F, *F*_(1,8)_ = 6.65, *p* < 0.05). *Post hoc* analyses of each interaction revealed a sensitivity to outcome devaluation for rats trained with pellets, but not for rats trained with sucrose.

These results suggest that rats trained with sucrose 20% progressively developed habitual responding while rats trained with pellets remained goal-directed. Because caloric intake was higher during pre-feeding with pellets compared to sucrose in this experiment (Supplementary Figure S2A, main effect of pre-feeding reward: *F*_(1,8)_ = 71.48, *p* < 0.0001), the unbalanced consumption of these rewards during the test may create a bias in general satiety and explain the dissociation observed in the results depicted in Figure 2. Indeed, for rats trained with sucrose, higher general satiety due to pre-feeding with pellets may reduce motivation in the valued condition and, thus, the difference between valued and devalued conditions, resulting in apparent insensitivity to outcome devaluation.

**Figure 3:**
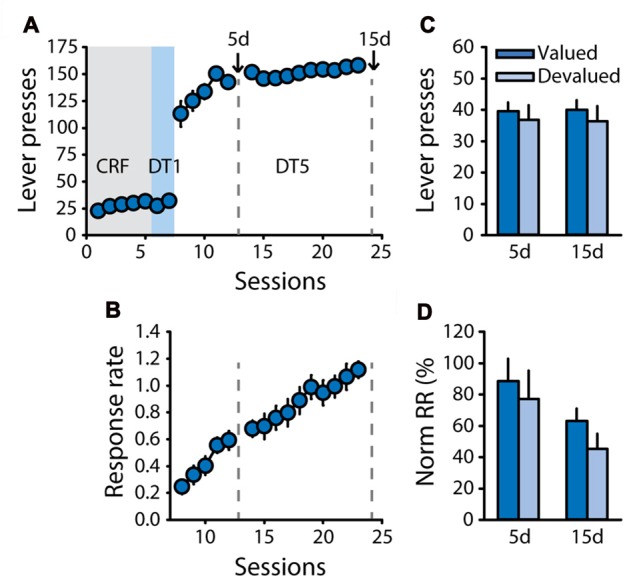
Development of habit-like responding for sucrose is not related to unbalanced caloric intake during pre-feeding. Training and satiety-induced devaluation tests in the DT5 procedure in rats trained with a 20% sucrose solution using a 20% maltodextrin solution as the control reward during pre-feeding for devaluation tests (*n* = 11). **(A)** Mean number of lever presses (±SEM) across training under CRF (five sessions), DT1 (two sessions) and DT5 (15 sessions) schedules in rats trained with sucrose 20%. **(B)** Mean response rate in responses per second (±SEM) across training under DT5. Gray dotted lines in **(A,B)** indicate the time of devaluation tests 5d and 15d. **(C)** Mean number of lever presses (±SEM) in valued (dark color; pre-fed with maltodextrin) and devalued (light color; pre-fed with sucrose) conditions across tests 5d and 15d. **(D)** Mean normalized response rate (±SEM) in valued (dark color) and devalued (light color) conditions across tests 5d and 15d.

Therefore, to control for any bias in general satiety during pre-feeding, a new cohort of rats (*n* = 11) was trained with sucrose 20% and was pre-fed with sucrose 20% or an isocaloric unsweetened solution of maltodextrin 20% (polycose) as the control reward during the devaluation test (Experiment 3). As expected, rats successfully learned the DT5 task, as indicated by their high level of lever pressing and progressive increase in response rate across sessions (Figures 3A,B, main effect of session on response rate, *F*_(14,140)_ = 29.57, *p* < 0.0001). Sensitivity to outcome devaluation was tested after 5 and 15 DT5 sessions. Caloric intake during pre-feeding access was similar in the valued and devalued conditions for both tests (Supplementary Figure S2B). Lever pressing and normalized response rates were not reduced in the devalued condition compared to the valued condition (Figures 3C,D), thus generally replicating results from Experiments 1 and 2, in absence of bias in general satiety. There was a significant effect of test on normalized response rate (*F*_(1,10)_ = 5.09, *p* < 0.05) but no devaluation by test interaction (all *F* values <0.3, ns).

### Tests of Contingency Degradation and Outcome Devaluation by Conditioned Taste Aversion Indicate Habitual-Like Responding for Both Sucrose and Pellet Rewards

**Figure 4:**
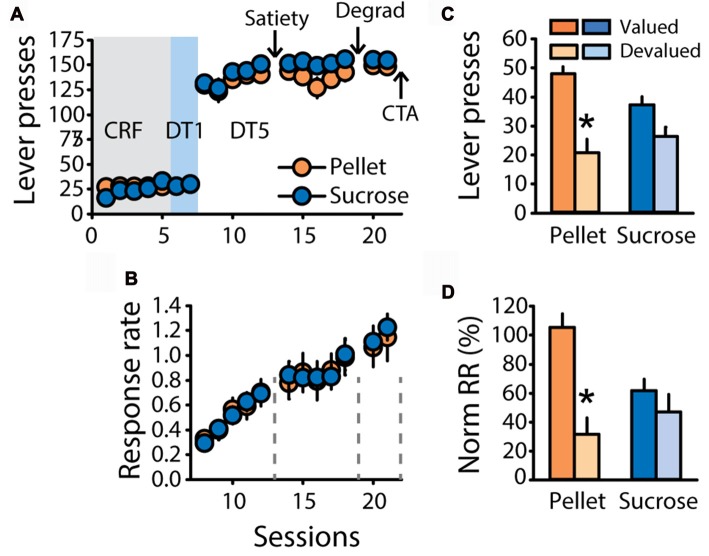
Replication of the findings depicted in Figure 2. **(A)** Mean number of lever presses (±SEM) across training under CRF (five sessions), DT1 (two sessions) and DT5 (12 sessions) schedules in rats trained with grain-based pellets (*n* = 11; orange circles) or sucrose (*n* = 12; blue circles). **(B)** Mean response rate in responses per second (±SEM) across training under DT5 in rats trained with pellets (orange circles) or sucrose (blue circles). Arrows in **(A)** and gray dotted lines in **(B)** indicate the time of satiety-induced devaluation (“Satiety”), degradation of contingency (“Degrad”) and devaluation by conditioned taste aversion (“CTA”). **(C)** Mean number of lever presses (±SEM) in the valued (dark color) and devalued (light color) conditions of the satiety-induced devaluation test, for rats trained with pellets (orange bars) or rats trained with sucrose (blue bars). *p < 0.001 compared to valued. **(D)** Mean normalized response rate (±SEM) in the valued (dark color) and devalued (light color) conditions of the satiety-induced devaluation test, for rats trained with pellets (orange bars) or rats trained with sucrose (blue bars). *p < 0.001 compared to valued.

To further investigate the differential sensitivity to outcome devaluation observed in the previous experiments, a new group of rats trained with grain-based pellets (*n* = 11) or 20% sucrose (*n* = 12) was tested for sensitivity to satiety-induced devaluation, contingency degradation and devaluation induced by CTA. As expected, learning of the DT5 task was associated with high levels of responding (Figure 4A) and a steady rise in response rate (Figure 4B, main effect of sessions *F*_(4,84)_ = 33.54, *p* < 0.0001) with no difference between groups (*F*_(1,21)_ = 1.32, ns). Sensitivity to satiety-induced devaluation was tested after five DT5 sessions, using the alternative reward as control reward during pre-feeding in the valued condition (pellets for rats trained with sucrose and reciprocally). As expected from the first two experiments, sensitivity to outcome devaluation interacted with the training reward (Figure 4C, lever presses: *F*_(1,21)_ = 7.62, *p* < 0.05, Figure 4D, normalized response rate: *F*_(1,21)_ = 8.41, *p* < 0.01). *Post hoc* analyses of these interactions revealed that lever pressing and normalized response rates were significantly lower in the devalued condition compared to the valued condition for rats trained with pellets (lever presses, *p* < 0.001, normalized response rate, *p* < 0.001). In contrast, these measures did not differ for rats trained with sucrose (*p* > 0.05).

Insensitivity to satiety-induced devaluation when rats are trained with sucrose could result from an absence of generalization of the devaluation from the feeding cage, in which rats drink in a bottle, to the operant box, in which rats lick the solution as it is delivered, in the receptacle. Alternatively, persistent sensitivity to satiety-induced devaluation when rats are trained with grain-based pellets could result from a bias in general satiety. Higher caloric intake, after pre-feeding with pellets in the devalued condition (Supplementary Figures S2A,C), could reduce hunger to a larger extent than pre-feeding with sucrose in the valued condition, creating an imbalance in motivational state during the test. To assess the response strategy of rats trained with pellets or sucrose in absence of bias related to pre-feeding conditions, this same group of rats was tested for degradation of contingency and devaluation by CTA.

**Figure 5:**
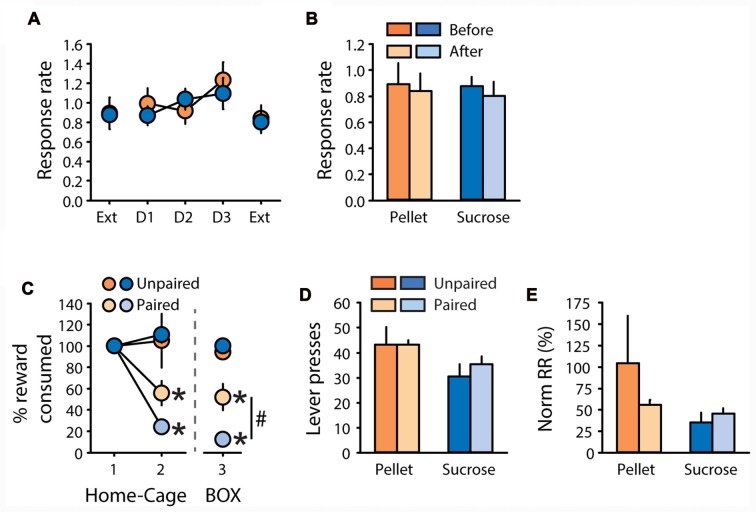
Subjects are insensitive to degradation of instrumental contingency and devaluation by CTA.** (A,B)** Contingency degradation **(A)** Mean response rate in responses per second (±SEM) during contingency degradation training and testing, in rats trained with grain-based pellets (*n* = 11; orange circles) or sucrose (*n* = 12; blue circles). On the *x*-axis, “Ext” indicates test sessions conducted under extinction before and after 3 days of contingency degradation training (D1, D2 and D3). **(B)** Mean response rate in responses per second (±SEM) before (dark color) and after (light color) contingency degradation training, in rats trained with grain-based pellets (orange bars) or sucrose (blue bars). **(C–E)** devaluation by CTA.** (C)** Reward consumption in the home cage during day 1 and 2 of taste aversion learning (“1–2”, “Home-Cage”) and during the test conducted on day 3 in the operant chamber (“3”, “BOX”). Consumption in the home-cage is expressed as the percentage of the amount eaten prior to lithium chloride treatment. Consumption in the operant box is expressed as the percentage of presented food that was consumed. Consumption was compared between subjects trained with pellets (orange circles) or sucrose (blue circles), in the group valued (unpaired: dark color) and devalued (paired: light color). *p < 0.0001 paired compared to unpaired. ^#^*p* < 0.01 paired sucrose vs. paired pellet on day 3. **(D)** Mean number of lever presses (±SEM) in valued (unpaired: dark color) and devalued (paired: light color) subjects during the devaluation test under extinction, for rats trained with pellets (orange bars) or sucrose (blue bars). **(E)** Mean normalized response rate (±SEM) in valued (unpaired: dark color) and devalued (paired: light color) subjects during the devaluation test under extinction, for rats trained with pellets (orange bars) or sucrose (blue bars).

Rats were tested in the contingency degradation procedure after 1 additional week of training. This procedure did not affect response rate, neither for rats trained with pellets nor for rats trained with sucrose (Figure 5A). Comparison of response rate during extinction tests before and after the degradation procedure did not reveal any effects of degradation, reward, or degradation by reward interactions (Figure 5B, all *F* values <2.1, ns). After two additional days of training, a CTA was induced. Pellets and sucrose were successfully devalued in paired rats compared to unpaired rats (Figure 5C). A two-factor ANOVA on the second consumption test revealed a significant effect of devaluation (*F*_(1,19)_ = 26.36, *P* < 0.0001) without effect of reward, nor a devaluation by reward interaction (*F* values < 2, ns). Importantly, the CTA readily generalized from the home-cage, where it was established, to the operant box, as indicated by a main effect of devaluation on sucrose and pellet consumption in the operant box (Figure 5C, “Box”, *F*_(1,19)_ = 77.92, *p* < 0.0001). In this consumption test on day 3, a devaluation by reward interaction (*F*_(1,19)_ = 9.39, *p* < 0.01) indicates that the sucrose solution was devalued to a larger extent than grain-based pellets (Figure 5C, “Box”, paired sucrose vs. paired pellets: *p* < 0.01). Despite devaluation of each training reward, paired rats did not show any reduction in lever presses (Figure 5D), or normalized response rate (Figure 5E), compared to unpaired rats (All *F* values < 1, ns). There was a significant effect of reward on lever pressing (Figure 5D, *F*_(1,19)_ = 5.33, *p* < 0.05) but no other significant main effects or interactions (all *F* values < 2.6, ns). Importantly, the insensitivity of pellet rats to devaluation by CTA cannot be explained by partial devaluation of grain-based pellets or additional training in the DT5 procedure between each test. Indeed, these results were replicated in another cohort of rats trained with pellets for five DT5 sessions. Despite the strong devaluation of their reinforcer, paired rats made similar numbers of lever presses with a similar response rate as unpaired rats (Supplementary Figures S3A–D), thus replicating results from this experiment.

### Responding for Sucrose Under a Free-Running Fixed-Ratio Schedule Is Sensitive to Outcome Devaluation

**Figure 6:**
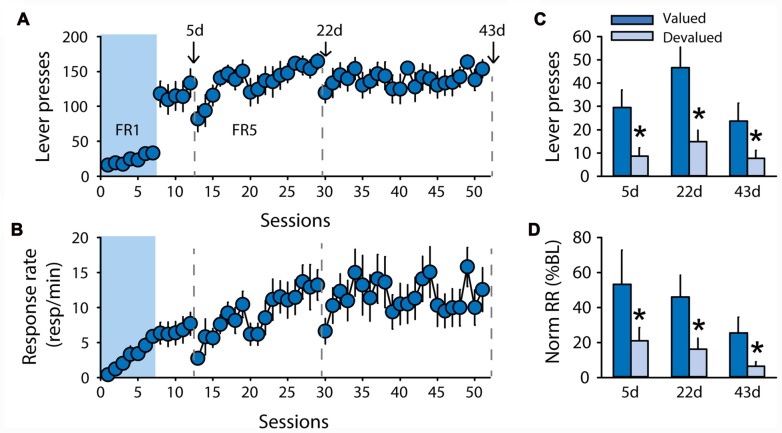
Subjects remain sensitive to outcome devaluation when trained to respond for sucrose under a free-running fixed ratio (FR) schedule. Training and satiety-induced devaluation tests in the FR5 procedure (*n* = 10). **(A)** Mean number of lever presses (±SEM) across training under CRF (seven sessions) and FR5 (43 sessions) in rats trained with 20% sucrose. **(B)** Mean response rate in responses per minute (±SEM) across training under CRF and FR5. Gray dotted lines in **(A,B)** indicate the time of devaluation tests 5d, 22d and 43d. **(C)** Mean number of lever presses (±SEM) in valued (dark color) and devalued (light color) conditions across tests 5d, 22d and 43d. *p < 0.01 compared to valued. **(D)** Mean normalized response rate (±SEM) in valued (dark color) and devalued (light color) conditions across tests 5d, 22d and 43d. *p < 0.01 compared to valued.

While the discrepancies in the results for rats trained with pellets raise important questions, the two devaluation procedures as well as the contingency degradation procedure suggest that rats trained with sucrose 20% in the DT5 procedure rapidly developed habitual responding. To test whether this insensitivity to outcome devaluation results from the structure of the DT5 procedure, an additional group of rats (*n* = 10) was trained with 20% sucrose under an FR5 schedule of reinforcement with continuous access to the lever (Experiment 5). Lever pressing and response rate progressively increased across sessions (Figure 6A, lever presses *F*_(43,387)_ = 1.52, *p* < 0.05, Figure 6B, response rate *F*_(43,387)_ = 2.34, *p* < 0.0001). Sensitivity to satiety-induced devaluation was tested after 5, 22 and 43 FR5 sessions. Grain-based pellets were used as the alternative (control) reward during the test to mimic the devaluation conditions of the second experiment. At each of the 3 tests, rats succeeded in modifying their responding according to the new outcome value, as indicated by a significant effect of devaluation on lever pressing (Figure 6C, *F*_(1,8)_ = 14.28, *p* < 0.01) and normalized response rate (Figure 6D, *F*_(1,8)_ = 20.55, *p* < 0.01). There was no main effect of test or a devaluation by test interaction (all *F* values < 3.7, ns).

### Quantitative Analyses Identify Differences in Within-Session Microstructure of Behavior Under Discrete Trials and Free Running Schedules

**Figure 7:**
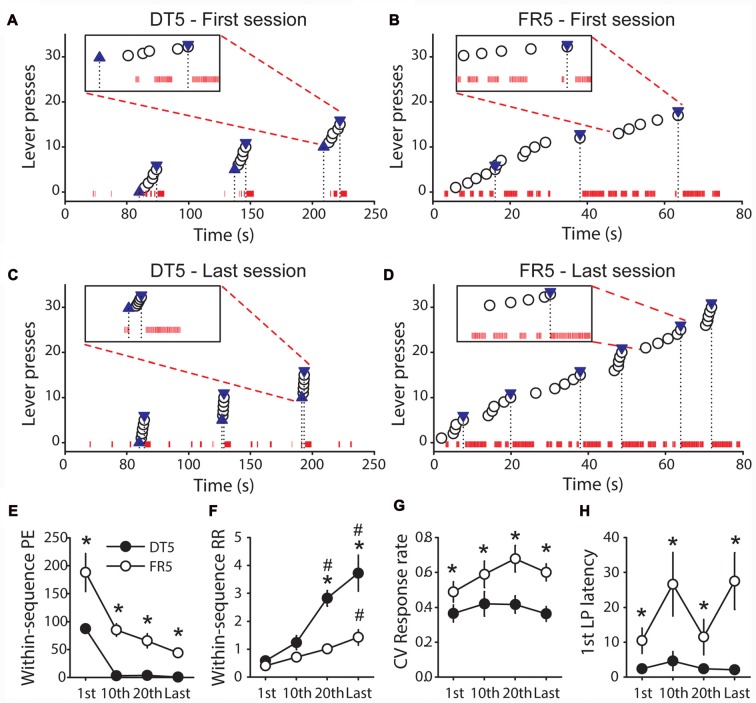
Comparison of performance between rats trained with sucrose in the DT5 and FR5 procedures. **(A,B)** Microstructure of the behavior of representative rats during the first trials of the first session of training in the DT5 **(A)** and in the FR5 **(B)** procedures. **(C,D)** Microstructure of the behavior of these same rats during the first trials of the last session of training in the DT5 **(C)** and in the FR5 **(D)** procedure. Each white circle indicates a lever press. In the DT5 procedure, blue upward triangles represent lever insertions and blue downward triangles represent lever retractions and reward deliveries **(A,C)**. In the FR5 procedure, downward triangles represent reward deliveries **(B,D)**. The black dashed lines indicate the time of these events. Red ticks on the *x* axis indicate rat presence in the magazine (one tick every 0.01 s of presence in the port). Inset in each figure shows the detailed microstructure during a specified trial along a scale of 20 s. **(E–H)** Group data. Average number of within-sequence port entries **(E)**, within-sequence response rate **(F)**, coefficient of variation of within-sequence response rate **(G)**, and 1st lever press latency **(H)** in the DT5 (black circles) and FR5 (white circles) procedures, during the 1st, 10th, 20th and last training sessions. Error bars denote SEM. Within-sequence response rate is expressed in responses per second. To allow comparisons of response rate between the DT5 and FR5 procedures, sequences exceeding 60 s in the FR5 procedure (time limit of lever availability in the DT5 procedure) were not considered. For the same reason, 1st lever press latencies exceeding 60 s in the FR5 procedure were set to 60 s. *P < 0.05, DT5 compared to FR5. ^#^*p* < 0.05, compared to 1st session.

The results above indicate that rats trained with sucrose in the FR5 procedure remained goal-directed whereas rats trained with the same reward in the DT5 procedure developed habit. To explore differences between these procedures we compared the microstructure of behavior and different measures of automaticity (within-sequence port entries, within-sequence response rate, response rate coefficient of variation and 1st lever press latency) at different points in training in Experiments 2 and 5. This analysis reveals that performance was more efficient in the DT5 procedure than in the FR5 procedure (Figures 7A–D). On the first day of training, visits of the port occurred frequently within and between the sequences of five lever presses for both procedures (Figures 7A,B). However, the number of within-sequence port entries drastically dropped after a few sessions in the DT5 procedure and did not significantly differ from zero on the 10th, 20th and last sessions (Figures 7C,E, all *t*-values < 2.2, ns). Within-sequence port entries also decreased in the FR5 procedure, but remained significantly above zero (all *t*-values > 5, *p* < 0.001) and higher compared to the DT5 procedure (Figures 7C–E, main effect of procedure *F*_(1,13)_ = 65.99, *p* < 0.0001, main effect of sessions *F*_(3,39)_ = 11.62, *p* < 0.0001). In addition, performance in the DT5 procedure was numerically greater and less variable than performance in the FR5 procedure (Figures 7A–D), as indicated by a main effect of procedure on within-sequence response rate (Figure 7F, *F*_(1,12)_ = 38.87, *p* < 0.0001) and on the coefficient of variation of within-sequence response rate (Figure 7G, *F*_(1,12)_ = 28.41, *p* < 0.001), respectively. Response rates increased to a larger extent over training in the DT5 procedure compared to the FR5 procedure (Figure 7F, interaction session*procedure: *F*_(3,36)_ = 8.49, *p* < 0.001). Finally, the 1st lever press latency was systematically shorter in the DT5 procedure (Figure 7H, main effect of procedure *F*_(1,13)_ = 6.86, *p* < 0.05).

## Discussion

The objective of this study was to determine whether the presence of a salient stimulus predictive of reward availability could promote the formation or expression of S-R habit. Our results demonstrate that providing this salient stimulus in the DT5 procedure promoted habitual responding in rats trained with 20% sucrose, as indicated by their insensitivity to outcome devaluation. Interestingly, rats trained with grain-based pellets remained persistently sensitive to satiety-induced devaluation, even after 8 weeks of DT5 training. This persistent sensitivity to outcome devaluation could result from a motivational bias during pre-feeding since rats trained with pellets were insensitive to contingency degradation and outcome-devaluation by CTA, suggesting that their behavior was in fact habitual. However, when sucrose was the reinforcer, the training schedule dictated whether responding was habitual or not. When rats were trained under the discrete-trials procedure, responding was insensitive to devaluation, and response measures were congruent with the development of a well-learned, automatic behavior. In contrast, when rats were trained under the more typical free-running fixed-ratio schedule, responding was sensitive to outcome devaluation, and response measures showed relatively greater trial-by-trial variability, congruent with a goal-directed behavior.

A previous study demonstrated that satiety-induced devaluation test results can vary depending on the ability of the reinforcer to induce satiety (Shillinglaw et al., 2014). Since pre-feeding with pellets generates higher caloric intakes than pre-feeding with sucrose, it could be argued that a bias in general satiety is responsible for the dissociation observed in this study. When the reinforcer is sucrose 20%, this bias is unlikely because our control experiment examining sensitivity to devaluation using sucrose and maltodextrin as pre-feeding rewards demonstrates habitual responding for sucrose despite similar caloric intake during pre-feeding access. Importantly, these two iso-caloric reinforcers activate different taste receptors and are distinguishable by rats (Nissenbaum and Sclafani, 1987; Sclafani, 2004), which makes them particularly suitable for induction of sensory-specific satiety. An absence of generalization of the devaluation from the feeding cage, in which rats drink in a bottle, to the operant box, in which rats lick the solution in the receptacle, could also explain the insensitivity of rats trained with sucrose to satiety-induced devaluation. However, this is unlikely because devaluation by CTA readily generalized from the home-cage to the operant box despite different drinking apparatus (bottle in home-cage vs. receptacle in operant box) and also revealed an insensitivity to outcome-devaluation. Although we did not assess specific-satiety in a consumption test after the devaluation sessions, the expression of goal-directed behavior in rats trained with sucrose in the FR5 procedure demonstrates that our pre-feeding protocol is sufficient for induction of devaluation via specific-satiety. Thus, together, results from multiple experiments of this study demonstrate that rats trained with 20% sucrose in the DT5 procedure rapidly developed habit.

When rats are trained with grain-based pellets, results are less consistent. More specifically, pellet rats were sensitive to satiety-induced devaluation, but insensitive to contingency degradation and devaluation by CTA. Although the contingency degradation test suggest that all the rats, whether trained with sucrose or pellets, expressed habitual behavior, it remains possible that the instrumental contingency was not degraded to a sufficient extent in our procedure to reveal a goal-directed behavior. Indeed, this procedure did not have any detectable effects on response rate, neither during degradation sessions nor during the test session under extinction. In absence of reliable goal-directed control, the efficacy of this procedure to degrade the contingency between the response and the outcome remains unclear. The outcome of this test—habitual responding for both training rewards—was however consistent with the general insensitivity to outcome devaluation induced by CTA. Importantly, the results for pellet rats cannot be explained by partial devaluation of grain-based pellets or additional training in the DT5 procedure between each test. Indeed, insensitivity to devaluation by CTA was replicated in another experiment after only 5 days of DT5 training with pellets, and with a more complete devaluation of the outcome. These results suggest that sensitivity to satiety-induced devaluation could results from a motivational bias during pre-feeding. Indeed, pre-feeding with pellets generated higher caloric intake than pre-feeding with sucrose. Furthermore, accumulating evidence in human suggests that ingestion of solid food generates higher satiation and satiety than beverages (Mattes and Rothacker, 2001; Zijlstra et al., 2008; de Wijk et al., 2008). Thus rats could be less hungry after pre-feeding with pellet compared to pre-feeding with sucrose. For rats trained with pellets, lower hunger in the devalued condition (pellets pre-feeding) compared to the valued condition (sucrose pre-feeding) could generate apparent sensitivity to the devaluation procedure. Additional experiments are needed to prove the existence of such bias in our experimental conditions. For example it would be interesting to determine the response strategy of rats trained with pellets using two types of pellet rewards during pre-feeding to ensure similar consumption and caloric intake in the valued and devalued conditions.

While additional experiments are necessary to demonstrate that responding for grain-based pellets is habitual, a clearer picture can be drawn from rats trained with sucrose. Formation of habit with this reinforcer occurred particularly fast in the DT5 procedure since the transition from goal-directed to habitual responding occurred between one and five training sessions in the first experiment. In the second experiment, lever pressing was still reduced in the devalued condition after 5 days of training and normalized response rate remained sensitive to devaluation after 5 and 17 days of training. The discrepancy in the time-course of habit development between Experiments 1 and 2 may result from a lack of reliability of satiety-based devaluation and suggests that results from studies using this approach should be interpreted cautiously. In subsequent experiments, performance was already habitual after 5 days of DT5 training. Considering that rats were trained under a ratio schedule known to promote goal-directed behavior (Dickinson et al., 1983), such a rapid formation of habit is surprising. Indeed, in a previous study from our laboratory, 8 weeks of training under random ratio 3 and exposure to ethanol were necessary to induce habit in rats trained with sucrose (Corbit et al., 2012). In fact, habitual responding after such limited training is typically observed in experiments using RI schedules (Lingawi and Balleine, 2012; Gremel and Costa, 2013) and/or drug self-administration (Barker et al., 2014; Loughlin et al., 2017), and does not fit well with the acknowledged habit formation emerging with extended practice. These results however could suggest an important role for reward-predictive stimuli in the formation and/or expression of S-R habit, and are consistent with previous studies demonstrating facilitated habit expression in presence of discriminative cues (Faure et al., 2005, 2010).

Results from the FR5 experiment indicate that the use of DT in the DT5 task may explain the insensitivity to outcome devaluation in rats trained with sucrose. More specifically, we showed that when the lever is continuously available in the FR5 procedure, rats remain goal-directed, whereas in the DT5 procedure, repeated lever insertions at each trial constitutes a salient stimulus predictive of reward availability that may promote habitual responding. These results thus confirm the important influence of stimuli on instrumental performance (Rescorla, 1987; Thrailkill and Bouton, 2015) and demonstrate their role in the expression of S-R habit. As noted earlier, conditioned stimuli can increase instrumental responding through PIT (Rescorla, 1994; Holland, 2004) and this PIT effect can counteract goal-directed behavior in humans by promoting cue-driven habitual responding (Watson et al., 2014; van Steenbergen et al., 2017). However, these PIT effects cannot account for the formation of habit in these studies because the stimulus and the response are associated with the outcome in separate training phases. In our study, since the stimulus (lever insertion/retraction) is present during instrumental learning, responding for sucrose can become habitual through the formation of S-R association. This does not preclude the possibility of a “PIT-like” effect; since this stimulus predicts reward availability and delivery, it can also form an association with the outcome. Stimulus presentations during devaluation tests could then lead to the expression of habit through PIT. To determine whether lever insertion promotes formation or expression of S-R habit, it would be interesting to run an experiment following a PIT procedure with separate S-O and R-O training phases and a devaluation test in presence of S, the stimulus S being the lever insertion. Habitual responding in these conditions would not be explained by the formation of S-R association since this association is never reinforced, and would therefore result from PIT. Whether our procedure promotes the formation or expression of habits, the current results suggest that salient Pavlovian stimuli can influence the control of instrumental performance by promoting habitual responding. Indeed, in the absence of salient reward-predictive stimuli (under FR5 schedule), responding for sucrose is goal-directed.

It is important to note that other factors may contribute to the expression of habit in the DT5 procedure, such as the trial structure, uncertainty about reward delivery, and the intervals between reward deliveries, all of which differ between the FR5 and DT5 procedures. For example, under DT5, predictions about reward availability and delivery through presentation and retraction of the lever respectively, allowed sequence learning and development of a strong automaticity, as evidenced by high response rates, low variability in performance, short lever press latencies and few within-sequence port entries. In the FR5 procedure, reward delivery was not signaled by lever retraction, which resulted in higher uncertainty, and less automaticity. Uncertainty in the FR5 procedure is well illustrated by higher numbers of within-sequence port entries, which presumably reflect the subjects’ checking for the presence of reward. Derusso et al. (2010) suggested that higher instrumental uncertainty promotes expression of habitual responding, which seems to be at odds with the results reported here. However, in Derusso’s study, the effect of instrumental uncertainty in the RI schedule can be mediated by weaker R-O contiguity, due to higher time intervals between each response and the reward delivery. This effect illustrates one limit of habit models using RI schedules; insensitivity to outcome devaluation may reflect the weaker strength of the R-O association established by the schedule, rather than the actual formation of S-R habit. In our study, uncertainty about reward delivery is higher in the FR5 procedure compared to the DT5 procedure but the contingency and contiguity between the response and the outcome are very strong, which likely results in a strong R-O association and expression of goal-directed behavior. The negative relation observed in our study between habit and instrumental uncertainty is however in agreement with neuro-computational models which posit that arbitration between goal-directed and habitual systems relies on the relative uncertainty of predictions from each system (Daw et al., 2005; Lee et al., 2014). Habit may be rapidly expressed in the DT5 procedure due to low uncertainty about predictions derived from the reward-predictive cues through reinforcement learning. Furthermore, strong automaticity and short lever press latencies in the DT5 procedure are consistent with the rapid development of habit observed in this study (Keramati et al., 2011).

Although automaticity and low uncertainty correlates with expression of habit in the DT5 procedure, it is important to note that the FR5 and DT5 experiments were run separately, which precludes strong conclusions about causality between these factors. Furthermore, instrumental performance can be both automatic (i.e., efficiency, discrimination, action chunking) and goal-directed (Derusso et al., 2010; Iguchi et al., 2017), suggesting that stronger automaticity is the DT5 procedure is not sufficient to explain the rapid development of habit observed in this study. Finally, although differences in automaticity of behavior are consistent with the opposite response strategies observed in the DT5 and FR5 procedures when rats are trained with sucrose, it cannot explain why rats trained with grain-based pellets in the DT5 procedure remained sensitive to satiety-induced devaluation (discussed above).

In summary, our study shows that providing a salient stimulus predictive of reward availability and delivery promotes the expression of S-R habits in rats trained with sucrose as indicated through traditional means of outcome devaluation (using sensory-specific satiety and CTA) and degradation of contingency, as well as through measures of behavioral automaticity. Although further experiments are necessary to explain inconsistencies in sensitivity to outcome devaluation for rats trained with pellets, these results demonstrate the strong influence of discrete reward-predictive cues on the control of instrumental performance. We propose, therefore, that a DT approach, in which the stimulus is readily identifiable and stimulus-triggered behavior can be quantified in detail, may be useful for probing the dependence of habits on the formation of S-R associations, and may ultimately allow for better definition of the place of habit in substance use disorders (Vandaele and Janak, 2017).

## Author Contributions

YV designed and carried out experiments, analyzed data and wrote the manuscript. HJP carried out experiments and participated in data extraction and analysis. PHJ designed experiments and wrote the manuscript. All authors critically reviewed content and approved the final version for publication.

## Conflict of Interest Statement

The authors declare that the research was conducted in the absence of any commercial or financial relationships that could be construed as a potential conflict of interest.
