# Supplementary material for: Lever Insertion as a Salient Stimulus Promoting Insensitivity to Outcome Devaluation
Source: Front Integr Neurosci. 2017 Sep 27;11:23. doi: 10.3389/fnint.2017.00023 (PMC5623688; doi:10.3389/fnint.2017.00023)
Supplement: Supplementary file 1 [file Data_Sheet_1.docx]

***SUPPLEMENTARY MATERIEL***

**Lever insertion as a salient stimulus promoting insensitivity to outcome devaluation**

**Youna Vandaele^1^, Heather Pribut^1^, Patricia H. Janak^1,2*^**

*** Correspondence:** Patricia H. Janak : [patricia.janak@jhu.edu](mailto:patricia.janak@jhu.edu)

# Supplementary Figure

- 1. **Supplementary figure 1**


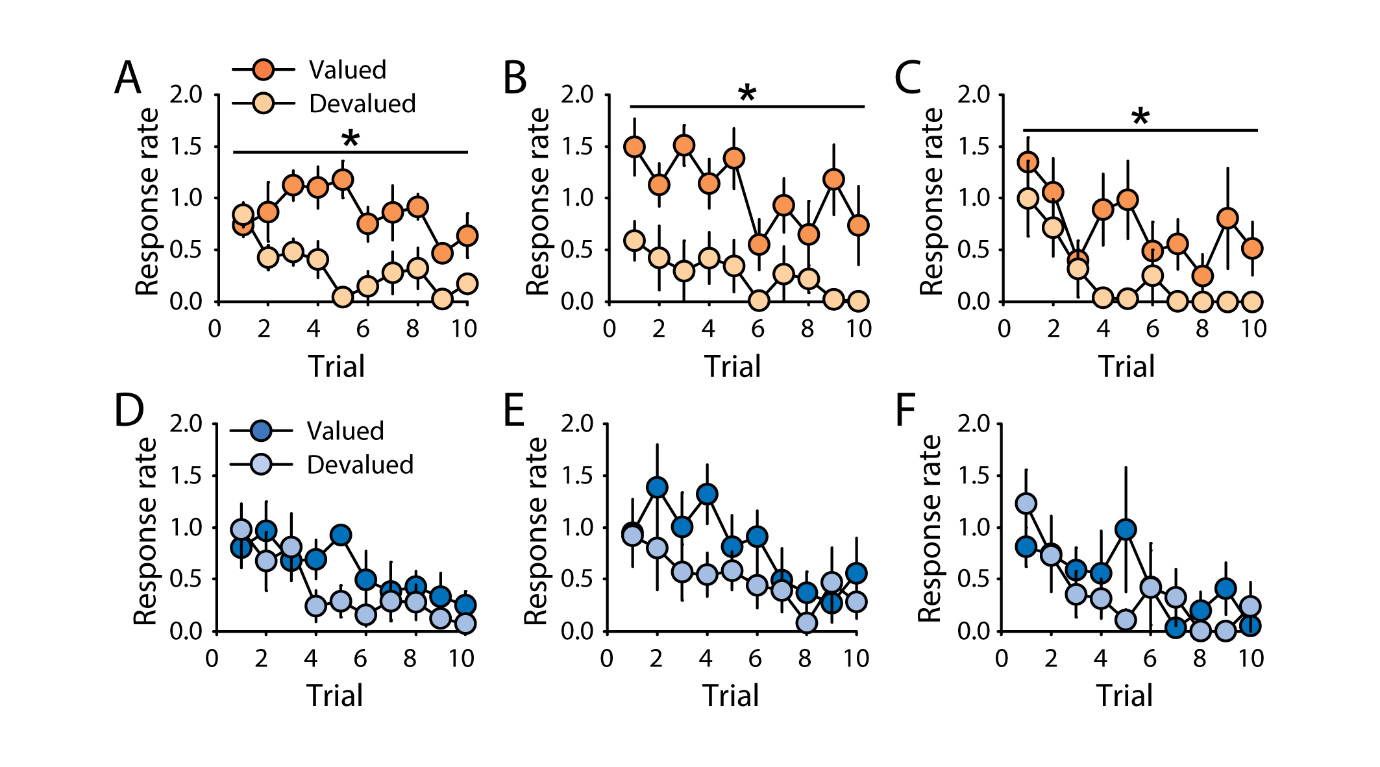


**Supplemental figure 1: Within-session responding during outcome devaluation tests for subjects depicted in Figure 2**. Response rate per trial in valued (dark color) and devalued (light color) conditions of devaluation tests 5d (**(A)** and **(D)**), 17d (**(B)** and **(E)**) and 43d (**(C)** and **(F)**), for rats trained with pellets **(A-C)** and rats trained with sucrose **(D-F)**. *p<0.05, devalued compared to valued.

- 1. **Supplementary figure 2**


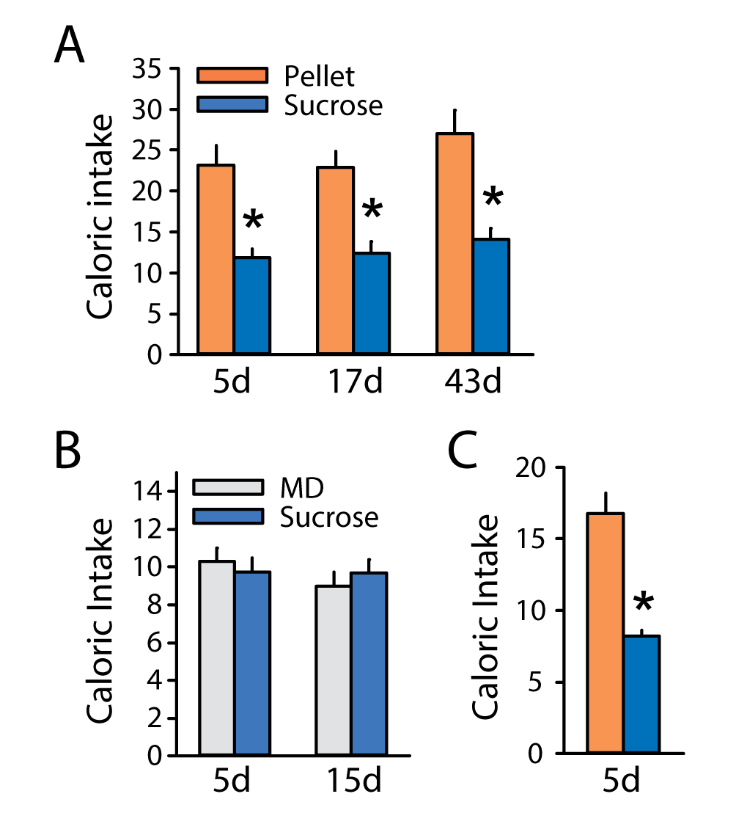


**Supplemental figure 2: Caloric intake during pre-feeding access of devaluation tests in experiments 2, 3 and 4.** **(A)** Caloric intake in kCal (±SEM) during pre-feeding with pellets (orange bars) or sucrose (blue bars) at tests 5d, 17d, and 43d of experiment 2. *p<0.0001 pellets compared to sucrose. **(B)** Caloric intake in kCal (±SEM) during pre-feeding with maltodextrin (grey bars) or sucrose (blue bars) at tests 5d and 15d of the experiment 3. **(C)** Caloric intake in kCal (±SEM) during pre-feeding with pellets (orange bar) or sucrose (blue bar) during the satiety-induced devaluation test of experiment 4. * p<0.0001 pellet compared to sucrose.

- 1.
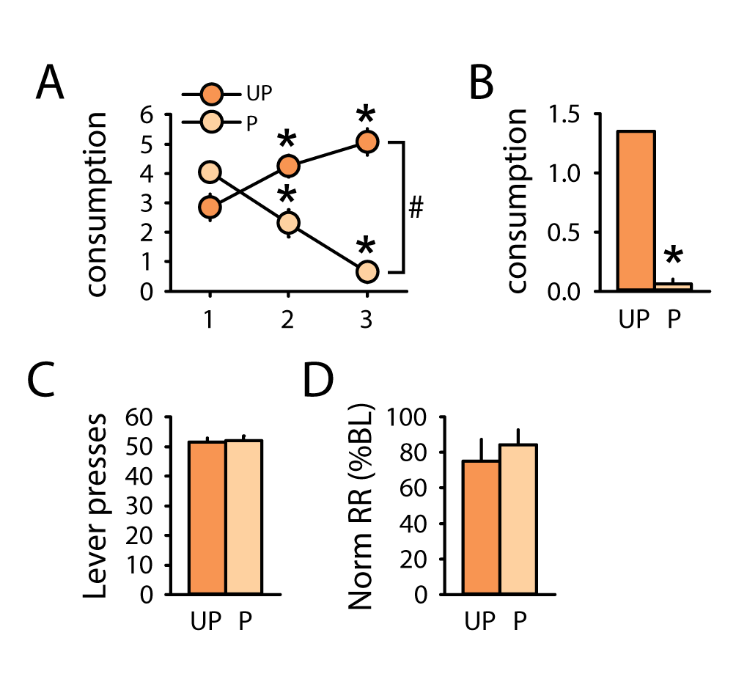
**Supplementary figure 3**

**Supplemental figure 3: Replication of the findings depicted in Figure 5, with rats trained with pellets and tested for their sensitivity to devaluation by conditioned taste aversion.** Rats previously trained to lever press for sucrose in the DT5 procedure under water deprivation, and tested for their sensitivity to outcome devaluation induced by conditioned taste aversion, were allowed to recover from water restriction and trained to respond for pellets in the DT5 procedure for 5 sessions under food restriction (90% of estimated free-feeding weight). These rats were then subjected to conditioned taste aversion. Neither CTA treatment altered responding during the extinction devaluation test. Results following CTA in food restricted subjects trained to respond for pellets are shown here. **(A)** Consumption (in g) during 10min free-feeding access to pellet in the home cage at days 1, 2 and 3 of taste aversion learning (‘1-2-3’) in valued (unpaired: dark orange) and devalued (paired: light orange) subjects. group effect F[1,10]=10.54, p<0.01, day effect F[3,20]=5.45, p<0.05, interaction group by day F[2,20]=115.90, p<0.0001. * p<0.001compared to day 1. #p<0.01 paired compared to unpaired on day 3. **(B)** Consumption (in g) during 5 min access to 30 pellets (max 1.35g) in the operant chamber in valued (unpaired UP: dark orange) and devalued (paired P: light orange) subjects. Group effect: t(10)=30.03, *p<0.0001 paired compared to unpaired. **(C)** Mean number of lever presses (±SEM) in valued (unpaired UP: dark orange) and devalued (paired P: light orange) subjects during the devaluation test under extinction. **(D)** Mean normalized response rate (±SEM) in valued (unpaired UP: dark orange) and devalued (paired P: light orange) subjects during the devaluation test under extinction.
